# Study protocol for developing an urban deprivation index in Nepal: Data review, measurement, visualization and real-world application in urban poverty alleviation

**DOI:** 10.1371/journal.pone.0324837

**Published:** 2025-06-11

**Authors:** Sampurna Kakchapati, Sitashma Mainali, Noemia Teixeira de Siqueira-Filha, Helen Elsey, Joseph Paul Hicks, Andrew Clark, Farzana Sehrin, Zahidul Quayyum, Bassey Ebenso, Sushil Chandra Baral

**Affiliations:** 1 H.E.R.D. International, Nepal; 2 Department of Health Sciences, University of York, United Kingdom; 3 University of Leeds, Leeds, United Kingdom; 4 University of Glasgow, Scotland; 5 BRAC James P Grant School of Public Health, BRAC University, Bangladesh; Public Library of Science, UNITED STATES OF AMERICA

## Abstract

**Background:**

Over the last two decades, Nepal has experienced substantial urbanization, with an increasing number of people choosing to move to cities. Although cities offer a wealth of opportunities, it also provides significant challenges. Many of those migrating to and living in cities contend with poor conditions and live in poverty. Defining and measuring urban deprivation is challenging due to its multi-dimensional nature, encompassing various dimensions such as housing, employment, living expenses, education, healthcare, and other unique challenges associated with city life. This study draws on the ‘Domains of Urban Deprivation Framework’ and evaluates the availability, commonness, and applicability of these domains. It measures multiple urban deprivation indices relevant to the context of Nepal.

**Method:**

The research will commence with a review of the availability of data covering the urban domains listed in the Urban Deprivation Framework within routine data collected in Nepal at the province, district, and municipal levels. This will involve examining existing datasets and identifying any gaps or limitations in the data that could impact the construction of the local urban deprivation indices. To understand the commonness of different urban deprivation within different urban contexts in Nepal, a Delphi survey will be conducted in two municipalities and nationally with government policymakers, community representatives, data experts/researchers, and civil society actors. In the three urban contexts, stakeholders will prioritize and weigh the indicators according to their respective urban contexts and rank domains that reflects the priorities across different geographical areas and stakeholder communities. We will compare responses across these groups of stakeholders and explore contextual differences. The composite score for each domain will be calculated by summing the weighted scores of all indices and normalizing the results to ensure that they fall within a defined range. We will then plot the deprivation indices in urban areas at the provincial, district, and municipal levels. The urban deprivation index in Nepal will provide granular data that will enable policymakers and stakeholders to explore the urban deprivation index visually and access key insights for informed decision-making and resource allocation.

**Ethics and dissemination:**

Ethical approval was obtained from the Ethical Review Board of Nepal Health Research Council (Reference number: 213/2024) and the School of Medicine Research Ethics Committee at the University of Leeds, UK. The findings will be disseminated in a peer-reviewed journal and presented at conferences.

## 1. Introduction

Urbanization is rapidly increasing, with more than half (55%) of the global population living in urban areas, according to the World Bank estimates [1]. This trend is expected to continue, with models estimating that 68% of the global population will be urban by 2050 [2,3]. Nepal is following global trends and facing a significant transformation driven by rapid urbanization. Historically, Nepal was predominantly rural, with a large majority of its population residing in villages and small towns. However, in recent decades, there has been a dramatic shift towards urban living [4]. The urbanization trends in Nepal are characterized by the expansion of existing urban centers and the emergence of new urban areas. According to the Central Bureau of Statistics, two-thirds of the Nepalese population (66%) lived in urban areas in 2021 [5]. This rapid urban growth has led to a surge in the urban poor population, exacerbating health disparities and straining already limited resources in urban areas [6,7]. This phenomenon poses significant challenges to urban environments, as inadequate access to necessities and services among this demographic amplifies existing social disparities and strains local infrastructure [3,8].

Thus, the urban poor constitute a vulnerable demographic facing a myriad of socio-economic hardships. To implement effective interventions, it is imperative to first understand the intricacies of their circumstances. However, this task is hindered by a critical gap in reliable and up-to-date data on urban poverty [8,9]. The absence of accurate information not only impedes our ability to grasp the full extent of the issues faced by the urban poor but also hampers the formulation of targeted policies and initiatives needed to uplift these marginalized communities. Moreover, the reliable and up-to-date data on urban poverty is spread across different datasets hindering evidence-based decision-making and progress towards the Sustainable Development Goals (SDGs) related to poverty reduction and urban development [8–10].

Defining the urban poor is challenging due to complex and dynamic factors inherent to urban environments [11]. Data on urban poverty is often constrained by challenges in accurately identifying and categorizing this demographic and difficulties in accessing marginalized urban areas for comprehensive surveys and assessments [12–14]. Additionally, varying definitions and thresholds for poverty across different regions and countries further complicate efforts to assess the data on urban poverty worldwide. Urban poverty and deprivation have been defined in diverse ways across various studies. Methods to measure poverty, including the Gini Coefficient, The World Bank’s Poverty Headcount Ratio, the Poverty Gap Index, and the Sen Index, among others, have been developed [11,15].

Through a scoping review, a framework has been developed to provide a high-level guide for identifying domains and indicators to measure urban deprivation. While this framework offers a comprehensive understanding of critical non-income factors—such as healthcare, education, housing, basic services, and infrastructure—associated with urban poverty, it does not provide specific methodologies or statistical techniques for constructing an index [11]. Building on this framework, we intend to develop an Urban Deprivation Index. This index will integrate the identified indicators to capture the multidimensional nature of urban challenges, offering a more comprehensive understanding compared to traditional income-based poverty measures. Methodologies for combining these indicators into a cohesive index will be explored in subsequent studies, drawing on relevant literature and established practices for index creation.

Furthermore, an alternative and more comprehensive version of an index has been devised through a scoping review, enabling the identification of poverty through a more streamlined set of variables. The urban deprivation index will provide a more comprehensive and multidimensional understanding of urban challenges compared to simply measuring urban poverty relying on income. The deprivation index will capture non-income related factors such as healthcare, education, housing, basic services, and infrastructure, which are critical in urban contexts and associated with poverty [11]. Additionally, the urban deprivation Indices along with global Sustainable Development Goals (SDGs), particularly SDG 11, by promoting inclusive, safe, and sustainable urban growth.

**Fig 1:**
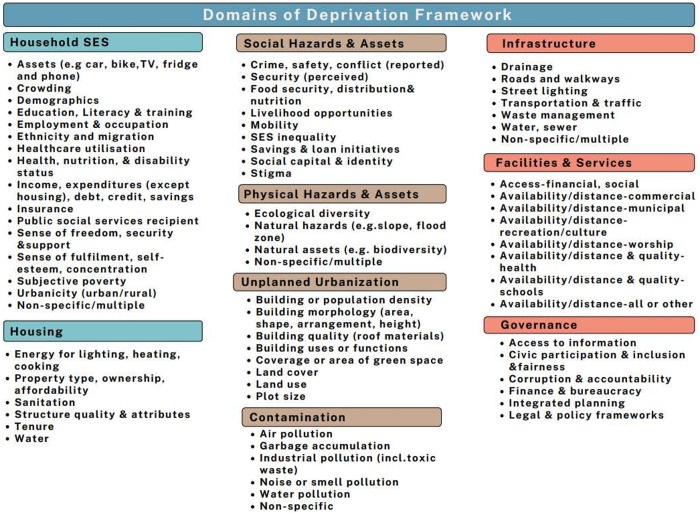
Domains of Deprivation Index [11].

In Nepal, where rapid urbanization is creating significant disparities, an urban deprivation index can provide more accurate insights for addressing socio-economic inequalities and improving the quality of life for the urban poor, supporting evidence-based policies for inclusive urban development [13]. In light of this, the project aims to adopt a widely recognized urban deprivation framework [11,12] to develop an urban deprivation index that is relevant to the urban context in Nepal. The index encompasses key domains such as socio-economic factors, housing, and environmental conditions, as shown in Fig 1.

This approach aims to provide a more comprehensive understanding of how to assess context-specific urban deprivation using existing data sets to equip policymakers and urban planners with a targeted tool to direct resources and interventions where they are most urgently required. The study will develop a user-centric urban deprivation index built in collaboration with government stakeholders.

## Method

### Availability of the urban deprivation domains in national datasets

#### Data assessment.

The research will begin by identifying and compiling potential data sources in Nepal to support the development of an urban deprivation index in Nepal, guided by the urban deprivation domains framework. The focus will be on exploring available data that align with the framework’s suggested indicators, enabling the construction of a comprehensive index that captures multidimensional aspects of urban deprivation. This process involves identifying and accessing datasets from various routine sources, including government agencies, non-governmental organizations (NGOs), academic institutions, and international organizations including the national surveys and routine health information management system. When selecting data sources, we will prioritize datasets that are part of established routine systems due to their reliability and likelihood of continued collection over time. We recognized that incorporating data that is infrequently collected or available only intermittently could pose challenges to the sustainability and regular computation of the index. To address this, we have adopted a systematic approach during the data mapping and exploration phase to assess the frequency, quality, and long-term availability of potential data sources. Key considerations include evaluate the periodicity, consistency, and likelihood of continued collection for each dataset to ensure the feasibility of its inclusion and wherever possible, we rely on routine data sources such as HMIS and national surveys, which offer greater reliability for ongoing data collection.

For critical indicators where, routine data is unavailable or infrequent, we explore alternative strategies such as using proxy indicators or modeled estimates. Subsequently, a comprehensive evaluation of available data sources will be conducted to assess their appropriateness for calculating the selected domains, considering factors like data quality, coverage, and relevance to local socio-economic conditions. This assessment will be conducted with a specific focus on their suitability for calculating the envisaged urban deprivation indices. Moreover, the process will involve an examination to uncover any gaps, limitations, or challenges in data collection and reporting. This assessment aims to ensure that the selected data sources are optimally aligned with the objectives of constructing accurate and meaningful urban deprivation indices.

Table 1 shows the information from census and national surveys and pertinent data sources from where the data will be compiled. When the deprivation domains are available in different datasets, we will adopt the following strategy (a) choose the survey data that is most representative of urban data, (b) use the most recent dataset, within five years, to ensure that the analysis reflects current urban deprivation dynamics, (c) use datasets including domains of urban deprivation index disaggregated by district, provincial and municipal levels; (d) use datasets providing high-quality data in terms of correctness, completeness, availability, and national representation and availability, including considerations for long-term availability as a routine data source to ensure future applicability. To enhance consistency across the indices, we will use standardized scales and methodologies where possible. For instance, if a specific scoring system is applied in one domain, it is replicated across others, ensuring methodological uniformity. This approach enhances the comparability and coherence of the indices while maintaining the integrity of the underlying data.

**Table 1:** Review of National survey and data sources reviewed for inclusion of Urban Deprivation Index.

Name	Year	Relevant Topics/Indicators	Appropriateness	Rank	Strength	Level
Nepal Population and Housing Census	2021	Population demographics, Housing conditions, Migration	Essential for population data, demographic trends	1	Conducted every 10 years, most comprehensive for population statistics	Federal, Provincial, District and Municipal
Nepal Living Standards Survey (NLSS)	2023	Poverty, Income, Living Conditions	Best for socio-economic data and poverty analysis	2	Most comprehensive poverty-related survey	Federal and Provincial
Survey of Nepali People	2022	Political opinions, Social behavior, Public attitudes	Excellent for understanding public opinion and behavior	3	Focused on societal trends and public sentiment; valuable for social research	Federal and Provincial
Nepal Demographic and Health Survey (NDHS)	2022	Health indicators, Maternal & Child Health, NCDs	Comprehensive health data, especially for maternal/child health	4	Regularly conducted, nationally representative	Federal and Provincial
Multiple Indicator Cluster Survey (MICS)	2019	Child health, Education, WASH	Good for tracking child development and basic indicators	5	International standards used, focused on child and women’s health	Federal and Provincial
Nepal Labour Force Survey (NLFS)	2018	Employment, Unemployment, Informal Sector	Best for labour market information	6	Source for robust and useful employment data may need supplementation for newer trends	Federal and Provincial
Nepal Health Facility Survey (NHFS)	2021	Health Infrastructure, Service Delivery	Appropriate for health system assessments	7	Useful for assessing health facility readiness and service quality	Federal and Provincial
Health Information Management System (HMIS)	Routine data	Healthcare access, service utilization	Focuses on health services data	8	Primarily health-centric, contributing to healthcare utilization	Federal and Provincial
Logistics Information Management System (LMIS)	Routine data	Availability and access to essential medical supplies	Focused on supply chain management	9	Useful for healthcare access and access to healthcare resources	Federal and Provincial

Based on an initial review, the Population Census (2021) emerged as the most comprehensive and reliable source of data and variables relevant to the urban deprivation index. We acknowledge that the Population Census is collected only once every 10–11 years, which raises concerns about its utility for regular updates to the index. This trade-off between data quality and frequency has been carefully considered. Despite its infrequent collection, the Census offers unparalleled depth and breadth of data that contributes major contribution to development of urban poverty index. This was followed by the National Living Standard Survey (NLSS) 2023 and the Survey of Nepali People 2022, both of which provided valuable insights on the urban deprivation index, though with slightly less coverage compared to the Population Census.

### Reviews of data sources for construction of the urban deprivation indices

This phase begins with the review of domains of deprivation index built in a previous scoping review [11] on deprivation framework for mapping deprived urban areas in LMICs (Figure 1). Based on the domains of deprivation index, a compilation of the data for each domain from the available data sources (Table 1) will be conducted. For example, the indicators for household socio-economic status include household amenities/assets or wealth, crowding, demographics, education, literacy and training, employment and occupation, ethnicity and migration, migration, health care utilization, health, nutrition, and disability status and Income, expenditure (except housing), debt, credit and savings. So, we will map the availability of these indicators in the datasets listed in Table 1. For each domain of the deprivation index, we will select their indicators, disaggregated by province/district/municipal.

### Appropriateness of the urban deprivation domains in the urban Nepal

#### Constructing an urban deprivation index: data and indicators.

After compiling data relevant to the diverse domains for the urban deprivation index, the development of a local deprivation index comprises of synthesis of information. The compilation of data for each domain will be conducted involving the extraction of specific indicators from each domain of the deprivation index. The finalization of indicator selection is guided by a comprehensive evaluation, considering data reliability, inclusivity, and alignment with policy goals. We will ensure reliability by selecting consistent indicators derived from census and national surveys, allowing for comparison. The wide range of indicators from each domain encompass diverse types of data, such as continuous, discrete, ordinal, multinomial, and binary variables. The normalization of data for the deprivation index involves standardizing continuous variables, categorizing and encoding categorical data, and scaling binary indicators to ensure comparability across diverse datasets (Table 2).

**Table 2:** Types of Data and Corresponding Normalization Methods for Deprivation Index.

Type of Data	Methods
Continuous Data (e.g., population, literacy rates, income)	Normalization Method: Min-Max Scaling or Z-score Normalization Min-Max Scaling: 𝑋′ = 𝑋 −𝑋 min 𝑋 max−𝑋 min Scales data to a range of 0–1. Z-Score Normalization: 𝑍 = 𝑋 − 𝜇 𝜎 Centers the data around 0 with a standard deviation of 1. Use this for normally distributed data.
Categorical Data (e.g., marital status, religion, occupation)	Normalization Method: One-Hot Encoding or Proportional Representation One-Hot Encoding: Creates binary columns for each category (e.g., “Single = 1, Married = 0”). Proportional Representation: Represent categories as proportions (e.g., percentage of the population in each category).
Binary Data (e.g., households with toilets, access to health insurance)	Normalization Method: No Change or Percentage Conversion Represent binary variables as percentages (e.g., “75% of households have toilets” as 0.75).
Composite Scores (e.g., Wealth Quintile, Social Security Allowance)	Normalization Method: Rescaling or Standardization Rescale to 0–1 if scores have a maximum and minimum range. Use Z-score normalization for derived scores (e.g., Wealth Index).
Spatial Data (e.g., ward-wise population, degree of urbanization)	Normalization Method: Relative Proportions or Area Weighting Normalize by area or population density. Use proportions forward-level data relative to the total (e.g., population of Ward A/Total Population).
Health Indicators (e.g., BMI, stunting, ANC services)	Normalization Method: Z-Scores for Standardized Metrics For child growth indicators (e.g., stunting, wasting): Use pre-defined Z-scores (e.g., WHO growth standards). Convert metrics like service coverage to percentages for comparability.
Infrastructure Data (e.g., road quality, housing materials)	Normalization Method: Ordinal Scaling or Binary Conversion If ordinal (e.g., poor, fair, good): Assign values (1 = poor, 2 = fair, 3 = good). For materials (e.g., concrete vs. mud): Use binary or proportional data.
Environmental Indicators (e.g., air quality, water quality)	Normalization Method: Index Scaling or Threshold Comparisons Index Scaling: Convert metrics like PM2.5 to a scale of 0–100 using defined thresholds. Threshold Comparison: Represent as percentages of compliance (e.g., % of days air quality met WHO standards).

The normalization process produces a standardized dataset where all indicators are transformed to comparable scales, ensuring consistency across diverse data types. This includes normalized values for continuous, categorical, and binary variables, making them ready for aggregation. These normalized indicators are then used to compute composite scores for each domain, summarizing the level of deprivation across different aspects.

### Agreement stage: delphi survey

To agree on the commonness of the urban deprivation indicators relevant to urban Nepal and assign weights to calculate the urban deprivation index, we will conduct a Delphi survey among Nepali stakeholders working on urbanization in developing countries. The Delphi method is a structured, iterative process designed to gather opinions from a panel of experts and achieve consensus through multiple rounds of surveys [15]. We will adapt the approach used by the COSMOS study, which applied a Delphi Survey to select a set of core outcomes for trials on multimorbidity.

We will carefully select and identify experts in relevant fields such as government municipal officials, urban planners, academics specializing in urbanization, representatives from NGOs working on urban development, and scientists familiar with deprivation indexes. To obtain a range of perspectives on urban deprivation, three distinct expert panels will be selected:

Group1: Government Municipal Officers from Pokhara Metropolitan City (PMC) and Budhanilkantha Municipality (BMC).

Group 2: Local NGOs and Civil Society Organizations (CSOs) operating in PMC and BMC.

Group 3: National Experts based in Nepal (but not international), including urban planners, social scientists, public health experts, and data analysts.

Each expert group will contribute their knowledge and expertise, but the focus on context-specific indicators will enable insights into how priorities differ between municipalities and at the national level. In this study, a three-round Delphi survey method will be employed to develop the urban deprivation index.

We will administer all surveys using the Delphi Manager V.5.0 platform. We will provide a lay description for each domain and indicator.

First Round: Assessing the inclusion and assigning weights to the urban deprivation indicators

In the first round, survey participants will be asked to score the importance of each indicator for inclusion in the construction of the Urban deprivation index in Nepal. The score will be based on their importance for measuring urban deprivation in their specific context (PMC, BMC, or national) without considering its feasibility or measurability. For scoring, the Grading of Recommendations Assessment, Development and Evaluations (GRADE) 9-point Likert scale will be used, with the following categories: ‘not important’ (scores 1–3), ‘important but not critical’ (scores 4–6) and ‘critical for inclusion’ (scores 7–9) (Table 3).

**Table 3:** Criteria for Categorization of domains/indicators in the Delphi Surveys.

Green	Critical for inclusion	Scores 7–9
Yellow	Important but not critical	Scores 4–6
Red	Not Important	Scores 1–3

Second Round: Reassessment

Participants will receive their own and the overall results (overall and for each stakeholder group) via email. They will be asked to re-score and re-weight the indicators using the same approach reported in the first round. In case of any change in scores or weights, participants will be asked to provide a reason for that in a free text. Results from round 2 will be analyzed and summarized following the same process as the first round.

Third round: Consensus meeting

The third round will be held via Zoom, and all participants will be invited to the consensus meeting via email. This round will reach a consensus on the green, blue, and yellow GRADE categories. Indicators classified as red GRADE will be automatically excluded from the discussion, documented with the rationale for their exclusion, and maintained in a supplementary list for potential future reassessment. Participants will receive the summary scores (overall and for each stakeholder group) before the meeting. During the meeting, we will present the study objectives and results of the second round. We will then ask participants to vote on the indicators into the categories ‘critical to include’ and ‘not important to include’. A similar process will be adopted for the weights, and participants will vote to agree or not (yes/no) with the average weight assigned for each indicator. Indicators classified as critical to include will be included if they meet the consensus meeting threshold of ≥80% voting for inclusion; otherwise, further discussion will occur until the agreement is reached.

### Applicability of the urban deprivation index: estimating the indices at province, district and municipal levels

The Delphi method will be employed to assign weights to each indicator based on expert consensus, allowing for an informed decision on how to combine the data into composite scores for each domain. Next, Principal Component Analysis (PCA) is applied to the dataset. PCA starts by constructing a matrix where each row represents units of analysis (e.g., neighborhoods or municipal or district) and each column represents normalized indicators. A covariance matrix is computed to identify relationships among the indicators, followed by calculating eigenvalues and eigenvectors. These eigenvectors define the principal components (PCs), which are linear combinations of the original indicators. The components that explain the majority of the variance in the data (e.g., 70%−90%) are retained, and their scores are calculated for each unit.

To align the PCA results with the Delphi survey outcomes, domain-specific weights are applied to the principal component scores, ensuring the index reflects expert judgment and contextual priorities. The weighted scores are aggregated to produce a single deprivation score for each unit of analysis. Finally, the index is validated by comparing it against known patterns of deprivation and conducting sensitivity analyses. The resulting Urban Deprivation Index provides multidimensional measure of deprivation, enabling rankings of neighborhoods or wards and highlighting spatial disparities through visualizations like maps or tables.

The urban deprivation index will be summarized to various provinces, districts, and municipalities using metrics such as mean or median deprivation scores. These measures will allow for comparisons between regions to identify areas with higher or lower overall deprivation. The index will also be analyzed at smaller spatial units (e.g., wards or neighborhoods) within municipalities to identify localized patterns of deprivation. This approach helps pinpoint specific areas within larger regions that may require targeted interventions. Using the R program, thematic maps will be generated to visually represent the deprivation index. These maps will use color gradients to show varying levels of deprivation, enabling clear visualization of spatial patterns and highlighting high-deprivation areas for targeted policy intervention and resource allocation.

The purpose of these visualizations is to highlight spatial patterns, allowing policymakers and stakeholders to identify areas with the highest deprivation and prioritize interventions accordingly. The analysis will present deprivation levels in an easily interpretable format, the maps will aid in evidence-based decision-making and equitable resource allocation. To ensure reproducibility, the mapping process will utilize open-source tools such as QGIS or R, with standardized data inputs and clear thresholds for color gradation. The methodology, including data sources and formatting requirements, will be provided as part of this resource, enabling other researchers and policymakers to adapt or replicate the visualizations in their own contexts. For example, maps could reveal areas with a lack of access to health facilities, prompting targeted healthcare infrastructure improvements.

### Capacity building and training

The capacity-building and training sessions with municipal officials constitute a vital aspect of the study, aiming to enhance their understanding and utilization of the developed urban deprivation index. During the training and capacity-building sessions, we will ensure that participants gain a thorough understanding of the index’s structure, methodology, and the significance of its outputs, such as deprivation scores and spatial visualizations. They will learn to interpret patterns and trends in the data, identify high-deprivation areas, and understand how these insights can inform targeted interventions and resource allocation. These workshops aim to provide participants with the skills and information needed to fully understand and apply the index, including appreciation of its limitations and maximizing its usefulness in urban planning and policymaking. The training includes instructions on how to use indexes, interpret data, and incorporate them into decision-making procedures. The focus is on establishing a cooperative atmosphere that motivates city officials to actively share their ideas and insights. The goal of the capacity-building sessions is to provide municipal officials with the knowledge and skills necessary to use the urban deprivation index to make informed decisions. This will enable them to address urban poverty more responsively and equitably by means of interactive discussions, workshops, and hands-on exercises.

### Study status and timeline

The study is currently ongoing, with several key phases completed or in progress. Participant recruitment for the Delphi survey, involving experts such as government municipal officers, urban planners, academics, NGO representatives, and scientists will be completed in April 2025.

Data compilation, which includes compiling datasets from national surveys and routine sources (e.g., Population Census 2021, NLSS 2023, HMIS), conducting the Delphi survey rounds, and gathering qualitative inputs from expert panels, commenced in January 2025 and is expected to be completed by June 2025. This timeline accounts for the iterative nature of the Delphi survey process (three rounds, including a consensus meeting) and potential logistical challenges in accessing and harmonizing diverse datasets from urban settings.

Data analysis, including normalization of indicators, Principal Component Analysis, and validation of the Urban Deprivation Index, will follow the completion of data collection. Results, including the finalized Urban Deprivation Index and associated visualizations (e.g., thematic maps), are anticipated to be completed by July 2025. These results will inform urban poverty alleviation strategies in Nepal and potentially other low- and middle-income countries.

### Strengthens and Limitations

#### Strengths.

- This study will develop context-relevant urban deprivation indices for use in Nepal, which has not been done before. This will provide novel insights and offer a new tool to assess and address urban poverty and inequality.
- The urban deprivation index calculation is generalizable, meaning it can be adapted and applied to other urban contexts in low- and middle-income countries (LMICs).
- It leverages data from census and national surveys, offering a robust data foundation for constructing the urban deprivation index.
- The results can directly inform decision-making and visualization, enhancing its practical utility for policy and planning.
- The index incorporates multiple domains of poverty, providing an enhanced coverage assessment of various aspects of urban deprivation.

#### Limitations.

- There may be disparities in the data compiled from national surveys, with some domains available at the district level and others at the provincial level, which could pose significant challenges to achieving uniformity in analysis and interpretation.
- Assigning appropriate weights to each domain of the index is complex, and variations in weighting given at the local level may impact the ability to produce one comprehensive index. However, the Delphi survey will address this problem as stakeholders will reach a consensus about the weights.
- The Delphi survey’s iterative consensus process allows expert-driven adjustments to indicator selection and weighting, leading to indices that reflect region-specific priorities but may vary across provincial, district, and municipal levels due to differing local contexts.
- The Delphi survey is a complex process and can affect the indices calculated at the provincial, district, and municipal levels.
- Another possible limitation is that the framework was built based on urban slum settlements. However, we are measuring the deprivation index based on overall urban poverty.

### Ethics and dissemination

This study primarily involved secondary data analysis and a Delphi survey with expert stakeholders in Nepal. For the secondary data analysis, all data were fully anonymized prior to access. The Delphi survey involved three expert panels: government municipal officers, local NGOs and civil society organizations operating in these municipalities, and national experts, including urban planners, social scientists, public health experts, and data analysts. All participants in the Delphi survey provided written informed consent prior to participation. Formal invitation letters were sent to participants, outlining the study’s purpose, the iterative three-round Delphi survey process, their role, potential risks and benefits, and their right to withdraw at any time. Participants were informed about the expected time commitment and the anonymized nature of their responses, which were collected to ensure confidentiality and encourage unbiased input. All data were stored securely in compliance with ethical guidelines. No minors were included in this study, as all participants were adult professionals. The study was approved by Ethical Review Board of Nepal Health Research Council (Reference number: 213/2024).

The study findings will be published in open-access journals to ensure that the findings are widely accessible to researchers, policymakers, and practitioners globally. The urban deprivation index will be shared with municipal governments, urban planners, and policymakers in Nepal through workshops and seminars to foster capacity building and informed decision-making. In addition to this, policy briefs will be developed and distributed to local authorities, government agencies, and non-governmental organizations working on urban planning and poverty alleviation, with practical recommendations for using the urban deprivation index to guide resource allocation.
